# Comparison of the value of various complex indexes of blood cell types and lipid levels in coronary heart disease

**DOI:** 10.3389/fcvm.2023.1284491

**Published:** 2023-12-15

**Authors:** Aihong Peng, Bing Zhang, Siyin Wang, Yujia Feng, Shengnan Liu, Cuiyi Liu, Shu Li, Fei Li, Yuanyuan Peng, Jing Wan

**Affiliations:** ^1^ Department of Cardiology, Zhongnan Hospital of Wuhan University, Wuhan, Hubei, China; ^2^ Department of Critical Medicine, Zhongnan Hospital of Wuhan University, Wuhan, Hubei, China

**Keywords:** coronary heart disease, Gensini score, SYNTAX score I, systemic inflammation-response index/high-density lipoprotein cholesterol count (SIRI/HDL), complex indexes

## Abstract

**Background:**

Inflammation and lipid infiltration play crucial roles in the development of atherosclerosis. This study aimed to investigate the association between various complex indexes of blood cell types and lipid levels with the severity of coronary artery stenosis and their predictive value in coronary heart disease (CHD).

**Methods:**

The retrospective study was conducted on 3,201 patients who underwent coronary angiography at the Department of Zhongnan Hospital of Wuhan University. The patients were divided into two groups: CHD group and non-CHD group. The CHD group was further classified into three subgroups (mild, moderate, severe) based on the tertiles of their Gensini score or SYNTAX score I. Various complex indexes of blood cell types and lipid levels were compared between the groups.

**Results:**

It revealed a positive correlation between all complex indexes and the severity of coronary artery stenosis. The systemic inflammation-response index/high-density lipoprotein cholesterol count (SIRI/HDL) exhibited the strongest correlation with both severity scores (Gensini score: *r* = 0.257, *P* < 0.001; SYNTAX score I: *r* = 0.171, *P* < 0.001). The monocyte to high-density lipoprotein cholesterol ratio (MHR) was identified as a stronger independent risk factor for CHD. However, SIRI/HDL had higher diagnostic efficacy for CHD (sensitivity 66.7%, specificity 60.4%, area under curve 0.680, 95% CI: 0.658–0.701). Notably, the pan-immune-inflammation value multiplied by low-density lipoprotein cholesterol count (PIV × LDL) exhibited the highest sensitivity of 85.2%.

**Conclusion:**

All complex indexes which we investigated exhibited positive correlations with the severity of coronary artery stenosis. SIRI/HDL demonstrated higher diagnostic efficiency for CHD and a significant correlation with the severity of coronary artery stenosis.

## 1. Introduction

The number of deaths attributed to non-communicable diseases (NCDs) worldwide in 2019 was 42.03 million, accounting for 74.36% of all deaths. Among NCDs, cardiovascular diseases accounted for the highest number of deaths, with coronary heart disease (CHD) being a major contributor (1). CHD accounted for 32.7% of the global burden of cardiovascular disease (2). Atherosclerosis, a sustained and dynamic inflammatory process in the vascular system, is a major underlying cause of CHD. It involves the interaction between immune responses and metabolic disturbances, leading to the formation and activation of coronary artery lesions (3, 4). Inflammatory cells (such as monocytes, neutrophils, and lymphocytes), lipid infiltration, platelet activation, coagulation cascade reactions, and platelet-fibrin clot formation play crucial roles in the development of atherosclerosis (5). In recent years, emerging indicators of inflammation and oxidative stress, such as the monocyte to high-density lipoprotein cholesterol ratio (MHR), neutrophil to lymphocyte ratio (NLR), systemic immune-inflammation index (SII), and systemic inflammation-response index (SIRI), have gained attention in assessing the severity and prognosis of CHD. Another parameter, the pan-immune-inflammation value (PIV) (6), has been defined as a comprehensive and cost-effective indicator of chronic low-grade inflammation. Bektas et al. found that PIV outperformed NLR and SII in predicting primary and secondary outcomes in ST-segment elevation myocardial infarction (STEMI) (7), with one-year all-cause mortality as the primary outcome. However, the correlation between PIV and the severity of coronary artery stenosis has not been explored in previous studies. In this study, we aimed to investigate the relationship between PIV and CHD while also examining complex indexes that combine leukocyte subtypes, platelet count, and lipid levels. We compared the diagnostic value of SIRI/HDL, SIRI × LDL, PIV/HDL, PIV × LDL, PIV, SIRI, SII, MHR, and NLR in predicting CHD and the severity of coronary artery stenosis.

## 2. Materials and methods

### 2.1. Study population and grouping

This retrospective study included a total of 3,201 patients who underwent coronary angiography at the Department of Cardiology, Zhongnan Hospital of Wuhan University, between January 2013 and December 2021. The diagnosis of coronary heart disease (CHD) was made based on the latest diagnostic criteria for coronary heart disease. Based on the results of coronary angiography, the patients were categorized into two groups: non-CHD group and CHD group. The non-CHD group consisted of 727 patients, including 401 males and 326 females. The CHD group comprised 2,474 patients, including 1,725 males and 749 females. Within the CHD group, further classification was performed based on the tertiles of either the Gensini score or the SYNTAX score I, resulting in three subgroups: mild, moderate, and severe.

### 2.2. Inclusion criteria

(1) With chest tightness, chest pain and other suspected coronary artery disease clinical manifestations; (2) Coronary angiography was performed and recorded; (3) With complete admission general data and relevant clinical examination data.

### 2.3. Exclusion criteria

(1) Various acute and chronic infectious diseases; (2) Hematologic diseases; (3) Surgical procedures and severe trauma within 3 months; (4) Autoimmune diseases or being treated with immunosuppressive drugs; (5) Malignant tumors; (6) Previous history of myocardial infarction; (7) Previous history of percutaneous coronary intervention (PCI) or coronary artery bypass grafting (CABG); (8) Cardiomyopathy or decompensated heart failure; (9) Severe hepatic and renal insufficiency; (10) Steroids use and incomplete clinical data.

### 2.4. Coronary angiography

Two experienced interventional cardiologists conducted coronary angiography and assessed the findings. The procedure involved multiple projections using the radial or femoral route, following the Judkins method. The cardiologists evaluated the degree of stenosis in the left main artery, left anterior descending artery, left circumflex artery, and right coronary artery. A stenosis of 50% or more in any of these coronary arteries can be diagnosed as coronary heart disease.

### 2.5. Gensini score or SYNTAX score I and grouping

Based on the results of the coronary angiography, the Gensini score and the SYNTAX score I were computed for each patient. In the coronary artery disease group, the patients were categorized into three groups based on the tertiles of the Gensini score. The groups were as follows: mild group (Gensini score < 20), moderate group (20 ≤ Gensini score ≤ 45), and severe group (Gensini score > 45). The SYNTAX score I was determined using the SYNTAX scoring website (https://syntaxscore.org/). For the patients with coronary heart disease, they were divided into three groups according to the tertiles of the SYNTAX score: mild group (SYNTAX score < 8), moderate group (8 ≤ SYNTAX score ≤ 16), and severe group (SYNTAX score > 16).

### 2.6. General information of patients and laboratory test results

General information of the patients was collected, which included their hospitalization number, age, gender, smoking status, hypertension, and diabetes mellitus. All patients underwent venous blood collection from the elbow in the early morning after admission, on an empty stomach (fasting for more than 10 h). The blood samples were tested by the Department of Clinical Laboratory at Zhongnan Hospital of Wuhan University. Various biochemical parameters were measured, including the following: neutrophil count (NEUT), lymphocyte count (LYMP), monocyte count (MONO), platelet count (PLT), white blood cell count (WBC), erythrocyte distribution width coefficient of variation (RDW-CV), total cholesterol (TC), triglycerides (TG), high-density lipoprotein cholesterol (HDL-C), low-density lipoprotein cholesterol (LDL-C), alkaline phosphatase (ALP), creatinine (Cr), Uric acid (UA), fasting glucose (FBG). These parameters were measured to assess the patients' biochemical profile and provide important information regarding their health status.

### 2.7. Composite indexes

PIV = [neutrophil count (×10^9^/L) × platelet count (×10^9^/L) ×  monocyte count (×10^9^/L)/lymphocyte count (×10^9^/L)]; SIRI = [neutrophil count (×10^9^/L) ×  monocyte count (×10^9^/L)/lymphocyte count (×10^9^/L)]; SII = [neutrophil count (×10^9^/L) ×  platelet count (×10^9^/L)/lymphocyte count (×10^9^/L)]; MHR = monocyte to high-density lipoprotein cholesterol ratio; NLR = neutrophil to lymphocyte ratio; SIRI/HDL = [SIRI/high-density lipoprotein cholesterol count(mmol/L)]; SIRI × LDL = [SIRI × low-density lipoprotein cholesterol count(mmol/L)]; PIV/HDL = [PIV/high-density lipoprotein cholesterol count(mmol/L)]; PIV × LDL = [PIV × low-density lipoprotein cholesterol count(mmol/L)].

### 2.8. Statistical methods

The measurement data were presented as mean ± standard deviation ($\bar{x}\pm s$) when they followed a normal distribution. For non-normally distributed measurement data, the median and interquartile range [M(Q3–Q1)] were used to describe the distribution. Count data were expressed as percentages (%). To compare two groups with normally distributed measures, the t-test was employed. When comparing multiple groups, the Analysis of Variance (ANOVA) was utilized. For conducting post-hoc comparisons between groups, the Least Significant Difference (LSD) analysis was employed. In cases where the measurement data did not conform to a normal distribution, the Mann-Whitney nonparametric test was employed for comparing two groups. For comparing multiple groups with non-normally distributed data, the Kruskal-Walli test was used. When dealing with count data, the *Χ*² test was employed for analysis. In the case of continuous variables following a normal distribution, Pearson correlation analysis was used. For variables that did not conform to a normal distribution, Spearman's rank correlation was employed. In the risk factor analysis of coronary heart disease, all variables underwent univariate Logistic analysis initially. Subsequently, composite variables showing statistical significance in the univariate analysis and other variables with statistical significance were subjected to multivariate Logistic regression analysis. The diagnostic efficacy was determined by employing Receiver Operating Characteristic (ROC) curve analysis. All statistical analyses were performed using the Statistical Package for the Social Sciences (SPSS) version 25 (IBM SPSS Inc., Chicago, USA). A significance level of 5% (*P* < 0.05) was considered statistically significant.

## 3. Results

### 3.1. Comparison of general information and composite indexes between the non-CHD group and the CHD group

It revealed that the majority of patients in the CHD group were male, accounting for 69.7% of the total. This suggests a higher prevalence of CHD in males compared to females. The prevalence of smoking, diabetes mellitus, and hypertension was significantly higher in the CHD group compared to the non-CHD group. Approximately 42.5% of patients with CHD were smokers, 27.8% had diabetes mellitus, and 63.8% had hypertension. These findings suggest that these factors contribute to the development of CHD and are more common among individuals with the disease. In terms of biomarkers and indices, the values of SIRI/HDL, SIRI × LDL, PIV/HDL, PIV × LDL, PIV, SIRI, SII, MHR, and NLR were higher in the CHD group compared to the non-CHD group. These biomarkers and indices represent different aspects of inflammatory immune responses and metabolic disturbances in the body. The observed differences in their values between the two groups indicate a potential association between these biomarkers and indices and the presence of CHD. These findings highlight the importance of considering these factors and biomarkers in the diagnosis and management of CHD (Table 1).

**Table 1:** Comparison of general information and complex indexes between the non-CHD group and the CHD group [($\bar{x}\pm s$), M (Q3–Q1), *n* (%)].

Indicators	Group		*P*-value
	Non-CHD group (*n* = 727)	CHD group (*n* = 2,474)	
Gender (male)	402 (55.2)	1,725 (69.7)	<0.001
Smoking	185 (29.0)	955 (42.5)	<0.001
Hypertension	372 (51.2)	1,578 (63.8)	<0.001
Diabetes mellitus	100 (13.8)	688 (27.8)	<0.001
Age (years)	59.96 ± 10.479	62.46 ± 10.280	<0.001
FBG (mmol/L)	5.38 (4.83–6.22)	5.53 (4.97–6.59)	0.005
WBC (×10^9^/L)	6.06 ± 1.71	7.13 ± 2.59	<0.001
LYMP (×10^9^/L)	1.71 ± 0.59	1.63 ± 0.59	0.002
MONO (×10^9^/L)	0.46 ± 0.35	0.54 ± 0.49	<0.001
NEUT (×10^9^/L)	3.81 ± 1.61	4.92 ± 3.45	<0.001
PLT (×10^9^/L)	196.64 ± 55.43	200.96 ± 56.96	0.071
RDW-CV (%)	13.15 ± 0.89	13.28 ± 1.32	0.001
ALP (U/L)	77.64 ± 37.33	80.52 ± 26.60	0.020
TC (mmol/L)	4.44 ± 1.09	4.38 ± 1.15	0.189
TG (mmol/L)	1.39 (1.01–2.04)	1.44 (1.03–2.08)	0.112
HDL (mmol/L)	1.19 ± 0.35	1.09 ± 0.33	<0.001
LDL (mmol/L)	2.67 ± 0.84	2.72 ± 0.92	0.215
Cr (mmol/L)	75.54 ± 29.35	76.72 ± 27.08	0.314
UA (mmol/L)	338.55 ± 94.30	358.58 ± 128.01	<0.001
SIRI/HDL	0.79 (0.52–1.22)	1.23 (0.76–2.094	<0.001
SIRI × LDL	2.36 (1.50–3.47)	3.25 (1.93–5.77)	<0.001
PIV/HDL	148.41 (92.90–251.18)	234.07 (140.48–434.58)	<0.001
PIV × LDL	471.48 (255.70–751.01)	624.84 (355.74–1175.25)	<0.001
PIV	171.26 (104.74–280.30)	246.01 (149.46–426.92)	<0.001
MHR	0.37 (0.27–0.49)	0.460 (0.34–0.63)	<0.001
NLR	2.14 (1.60–2.93)	2.62 (1.88–3.82)	<0.001
SII	408.18 (275.13–599.02)	514.18 (347.30–775.1)	<0.001
SIRI	0.92 (0.61–1.33)	1.27 (0.81–2.04)	<0.001

FBG, fasting blood glucose; WBC, white blood cell count; LYMP, lymphocyte count; MONO, monocyte count; NEUT, neutrophil count; PLT, platelet count; RDW-CV, erythrocyte distribution width coefficient of variation; ALP, alkaline phosphatase; TC, total cholesterol; TG, triglyceride; HDL, high-density lipoprotein cholesterol; LDL, low-density lipoprotein cholesterol; Cr, creatinine; UA, uric acid; PIV: pan-immune-inflammation value; SIRI, systemic inflammation-response index; SII, systemic immune-inflammation index; MHR, monocyte to high-density lipoprotein cholesterol ratio; NLR, neutrophil to lymphocyte ratio; SIRI/HDL, SIRI/high-density lipoprotein cholesterol count; SIRI × LDL, SIRI × low-density lipoprotein cholesterol count; PIV/HDL, PIV/high-density lipoprotein cholesterol count; PIV × LDL, PIV × low-density lipoprotein cholesterol count.

### 3.2. Comparison of general information and composite indexes among subgroups of coronary heart disease patients

The CHD group was further divided into mild, moderate, and severe categories based on the Gensini score tertiles. Comparing the complex indexes, it was observed that the severe group had higher values of SIRI/HDL, SIRI × LDL, PIV/HDL, PIV × LDL, PIV, SIRI, SII, MHR, and NLR compared to the other two groups. Additionally, the moderate and severe groups had higher values of SIRI/HDL, SIRI × LDL, PIV/HDL, PIV × LDL, PIV, SIRI, and NLR compared to the mild group. These differences were statistically significant, with a *P*-value of less than 0.05 (Table 2). Similarly, the CHD group was also categorized into mild, moderate, and severe groups based on the SYNTAX score I tertiles. In this analysis, the severe group exhibited higher values of SIRI/HDL, SIRI × LDL, PIV/HDL, PIV, SIRI, SII, MHR, and NLR compared to the other two groups. Moreover, the moderate and severe groups had higher values of SIRI/HDL, SIRI × LDL, PIV/HDL, PIV × LDL, PIV, SIRI, SII, and NLR compared to the mild group. These differences were statistically significant, with a *P*-value of less than 0.05 (Table 3). In summary, when categorizing the CHD group based on the severity of coronary artery lesions determined by the Gensini score and SYNTAX score I tertiles, the severe groups consistently exhibited higher values of various composite indexes compared to the mild and moderate groups. These findings suggest that these composite indexes are associated with the severity of coronary artery lesions and can potentially serve as indicators for assessing the extent and severity of CHD.

**Table 2:** Comparison of general information and complex indexes in the gensini score triple quartile group for CHD [($\bar{x}\pm s$), M (Q3–Q1), *n* (%)].

Indicators	Group			*P*-value
	Mild group (*n* = 779)	Moderate group (*n* = 873)	Severe group (*n* = 822)	
Gender (male)	508 (65.2)	611 (70.0)	606 (73.7)^a^	0.001
Smoking	264 (37.4)	354 (44.0)^a^	337 (45.7)^a^	0.004
Hypertension	498 (63.9)	552 (63.2)	528 (64.2)	0.907
Diabetes mellitus	175 (22.5)	251 (28.8)^a^	262 (31.9)^a^	<0.001
Age (years)	62.14 ± 9.86	62.35 ± 10.02	62.87 ± 1.92	0.341
FBG (mmol/L)	5.38 (4.92–6.10)	5.50 (4.93–6.52)^a^	5.74 (5.05–7.17)^a,b^	<0.001
WBC (×10^9^/L)	6.45 ± 2.04	7.08 ± 2.53^a^	7.83 ± 2.93^a^	<0.001
LYMP (×10^9^/L)	1.67 ± 0.59	1.64 ± 0.58	1.58 ± 0.611^a^	0.012
MONO (×10^9^/L)	0.48 ± 0.16	0.52 ± 0.33	0.61 ± 0.75^a,b^	<0.001
NEUT (×10^9^/L)	4.22 ± 3.06	4.86 ± 3.40^a^	5.66 ± 3.71^a^	<0.001
PLT (×10^9^/L)	198.69 ± 54.80	201.15 ± 56.38	202.92 ± 59.50	0.330
RDW-CV (%)	13.29 ± 1.08	13.24 ± 1.05	13.30 ± 1.72	0.650
ALP (U/L)	79.55 ± 26.55	79.76 ± 25.45	82.245 ± 27.78^a^	0.074
TC (mmol/L)	4.35 ± 1.09	4.34 ± 1.19	4.45 ± 1.17^b^	0.089
TG (mmol/L)	1.40 (1.00–2.01)	1.07 (1.49–2.18)	1.02 (1.43–2.08)	0.320
HDL (mmol/L)	1.13 ± 0.31	1.09 ± 0.36^a^	1.04 ± 0.30^a,b^	<0.001
LDL (mmol/L)	2.65 ± 0.89	2.68 ± 0.93	2.82 ± 0.93^a,b^	<0.001
Cr (mmol/L)	75.26 ± 22.51	76.57 ± 28.07	78.25 ± 29.79^a^	0.090
UA (mmol/L)	354.12 ± 97.53	354.30 ± 97.79	367.36 ± 173.41^a,b^	0.056
SIRI/HDL	1.04 (0.68–1.49)	1.18 (0.74–2.01)^a^	1.60 (0.94–2.89)^a,b^	<0.001
SIRI × LDL	2.61 (1.66–4.24)	3.02 (1.84–5.47)^a^	4.38 (2.45–7.90)^a,b^	<0.001
PIV/HDL	196.92 (125.57–310.18)	228.79 (133.20–228.79)^a^	300.16 (170.49–588.26)^a,b^	<0.001
PIV × LDL	518.91 (311.36–878.14)	588.63 (335.53–1125.88)^a^	811.38 (449.87–1598.04)^a,b^	<0.001
PIV	213.16 (136.12–322.98)	237.40 (144.64–435.09)^a^	305.69 (171.61–565.67)^a,b^	<0.001
MHR	0.43 (0.32–0.56)	0.46 (0.35–0.63)	0.50 (0.36–0.66)^a,b^	<0.001
NLR	2.30 (1.71–3.30)	2.57 (1.87–3.69)^a^	3.05 (2.12–5.12)^a,b^	<0.001
SII	470.20 (318.10–644.83)	497.17 (345.19–760.32)	593.26 (382.96–1032.66)^a,b^	<0.001
SIRI	1.10 (0.75–1.62)	1.20 (0.77–2.04)^a^	1.57 (0.98–2.74)^a,b^	<0.001

FBG, fasting blood glucose; WBC, white blood cell count; LYMP, lymphocyte count; MONO, monocyte count; NEUT, neutrophil count; PLT, platelet count; RDW-CV, erythrocyte distribution width coefficient of variation; ALP, alkaline phosphatase; TC, total cholesterol; TG, triglyceride; HDL, high-density lipoprotein cholesterol; LDL, low-density lipoprotein cholesterol; Cr, creatinine; UA, uric acid; PIV: pan-immune-inflammation value; SIRI, systemic inflammation-response index; SII, systemic immune-inflammation index; MHR, monocyte to high-density lipoprotein cholesterol ratio; NLR, neutrophil to lymphocyte ratio; SIRI/HDL, SIRI/ high-density lipoprotein cholesterol count; SIRI × LDL, SIRI × low-density lipoprotein cholesterol count; PIV/HDL, PIV/high-density lipoprotein cholesterol count; PIV × LDL, PIV × low-density lipoprotein cholesterol count.

^a^ *P* < 0.05 compared to the mild group.

^b^ *P* < 0.05 compared to the moderate group.

**Table 3:** Comparison of general information and complex indexes in the SYNTAX score triple quartile group for CHD [($\bar{x}\pm s$), M (Q3–Q1), *n* (%)].

Indicators	Group			*P*-value
	Mild group (*n* = 807)	Moderate group (*n* = 878)	Severe group (*n* = 787)	
Gender (male)	547 (67.8%)	601 (68.5%)	576 (73.2%)	0.037
Smoking	290 (39.7%)	343 (42.5%)	322 (45.4%)	0.093
Hypertension	507 (62.8%)	558 (63.6%)	512 (65.1%)	0.640
Diabetes mellitus	188 (23.3%)	251 (28.6%)^a^	249 (31.6%)^a,b^	0.001
Age (years)	61.13 ± 10.00	62.75 ± 10.09^a^	63.5 ± 10.64^a^	<0.001
FBG (mmol/L)	5.41 (4.93–6.12)	5.54 (4.95–6.53)	5.71 (5.04–7.12)^a,b^	<0.001
WBC (×10^9^/L)	6.76 ± 2.42	7.10 ± 2.42^a^	7.56 ± 2.88^a,b^	<0.001
LYMP (×10^9^/L)	1.66 ± 0.58	1.64 ± 0.58	1.58 ± 0.62^a,b^	0.018
MONO (×10^9^/L)	0.49 ± 0.18	0.53 ± 0.32	0.60 ± 0.77^a,b^	<0.001
NEUT (×10^9^/L)	4.44 ± 2.24	5.02 ± 4.66^a^	5.31 ± 2.78^a^	<0.001
PLT (×10^9^/L)	200.12 ± 57.25	201.94 ± 55.75	200.63 ± 58.06	0.793
RDW-CV (％)	13.23 ± 1.08	13.32 ± 1.69	13.28 ± 1.05	0.382
ALP (U/L）	80.54 ± 26.09	79.31 ± 27.56	80.50 ± 26.60	0.166
TC (mmol/L)	4.36 ± 1.11	4.39 ± 1.15	4.39 ± 1.20	0.857
TG (mmol/L)	1.45 (1.06–2.09）	1.47 (1.04–2.19)	1.40 (0.99–1.99)	0.575
HDL (mmol/L)	1.12 ± 0.33	1.09 ± 0.32	1.05 ± 0.33^a,b^	0.001
LDL (mmol/L)	2.68 ± 0.92	2.72 ± 0.88	2.75 ± 0.95	0.248
Cr (mmol/L)	76.03 ± 27.00	75.38 ± 23.88	78.91 ± 30.26^a,b^	0.021
UA (mmol/L)	352.84 ± 94.86	356.32 ± 100.27	366.93 ± 176.05	0.073
SIRI/HDL	1.07 (0.72–1.65)	1.23 (0.74–2.09)^a^	1.46 (0.89–2.57)^a,b^	<0.001
SIRI × LDL	2.87 (1.77–4.61)	3.20 (1.88–6.00)^a^	3.75 (2.20–7.01)^a,b^	<0.001
PIV/HDL	210.86 (133.62–344.73)	229.51 (136.59–445.61)^a^	265.72 (152.31–504.43)^a,b^	<0.001
PIV × LDL	553.33 (327.89–1031.95)	625.46 (352.67–1158.75)^a^	714.85 (395.64–1373.39)^a^	<0.001
PIV	215.80 (144.92–371.66)	249.10 (146.80–436.48)^a^	272.37 (158.81–503.31)^a,b^	<0.001
MHR	0.44 (0.33–0.58)	0.46 (0.34–0.63)	0.48 (0.36–0.65)^a,b^	<0.001
NLR	2.42 (1.79–3.46)	2.62 (1.87–3.80)^a^	2.91 (2.04–4.66)^a,b^	<0.001
SII	492.42 (335.97–693.40)	510.95 (345.49–771.01)^a^	544.52 (356.01–892.97)^a,b^	<0.001
SIRI	1.13 (0.77–1.71)	1.30 (0.78–2.10)^a^	1.45 (0.93–2.47)^a,b^	<0.001

FBG, fasting blood glucose; WBC, white blood cell count; LYMP, lymphocyte count; MONO, monocyte count; NEUT, neutrophil count; PLT, platelet count; RDW-CV, erythrocyte distribution width coefficient of variation; ALP, alkaline phosphatase; TC, total cholesterol; TG, triglyceride; HDL, high-density lipoprotein cholesterol; LDL, low-density lipoprotein cholesterol; Cr, creatinine; UA, uric acid; PIV: pan-immune-inflammation value; SIRI, systemic inflammation-response index; SII, systemic immune-inflammation index; MHR, monocyte to high-density lipoprotein cholesterol ratio; NLR, neutrophil to lymphocyte ratio; SIRI/HDL, SIRI/high-density lipoprotein cholesterol count; SIRI × LDL, SIRI × low-density lipoprotein cholesterol count; PIV/HDL, PIV/high-density lipoprotein cholesterol count; PIV × LDL, PIV × low-density lipoprotein cholesterol count.

^a^ *P* < 0.05 compared to the mild group.

^b^ *P* < 0.05 compared to the moderate group.

### 3.3. Correlation between complex index and gensini score or SYNTAX score I

**Figure 1:**
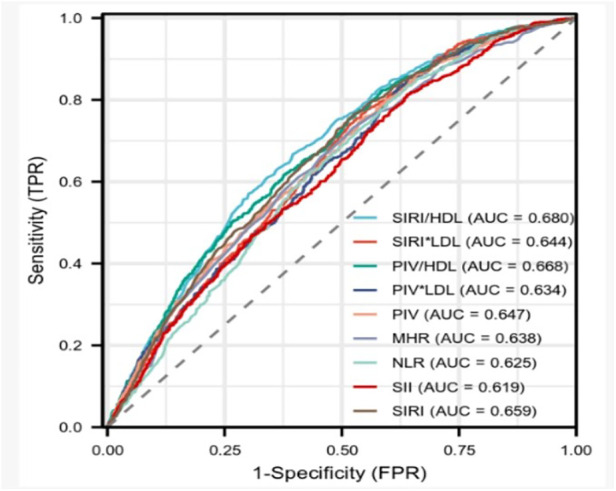
Receiver operating characteristic (ROC) curve analysis.

Spearman correlation analysis was conducted to examine the relationship between various factors, including SIRI/HDL, SIRI × LDL, PIV/HDL, PIV × LDL, PIV, SIRI, SII, MHR, NLR, and Gensini score or SYNTAX score I. The findings revealed that SIRI/HDL, SIRI × LDL, PIV/HDL, PIV × LDL, PIV, SIRI, SII, MHR, and NLR exhibited positive correlations with the Gensini score. Among these factors, SIRI/HDL demonstrated the strongest correlation with a coefficient of 0.257 (*P* < 0.001) (Figure 1). Additionally, SIRI/HDL, SIRI × LDL, PIV/HDL, PIV × LDL, PIV, SIRI, SII, MHR, and NLR were also positively correlated with SYNTAX scores I, although the correlation was relatively weak. SIRI/HDL again displayed the strongest correlation with a coefficient of 0.171 (*P* < 0.001) (Table 4). This analysis suggests that SIRI/HDL, SIRI × LDL, PIV/HDL, PIV × LDL, PIV, SIRI, SII, MHR, and NLR are associated with both Gensini score and SYNTAX score I, indicating their potential relevance to the severity of coronary artery disease. Notably, SIRI/HDL appears to have the strongest correlation with both scoring systems.

**Table 4:** Correlation of complex indexes with gensini score or SYNTAX score.

Indicators	Gensini score		SYNTAX score	*P*-value
	*r*-value	*P*-value	*r*-value	
SIRI/HDL	0.257	<0.001	0.171	<0.001
SIRI × LDL	0.240	<0.001	0.160	<0.001
PIV/HDL	0.243	<0.001	0.160	<0.001
PIV × LDL	0.222	<0.001	0.144	<0.001
PIV	0.212	<0.001	0.138	<0.001
SIRI	0.168	<0.001	0.110	<0.001
SII	0.214	<0.001	0.148	<0.001
MHR	0.200	<0.001	0.133	<0.001
NLR	0.228	<0.001	0.152	<0.001

PIV, pan-immune-inflammation value; SIRI, systemic inflammation-response index; SII, systemic immune-inflammation index; MHR, monocyte to high-density lipoprotein cholesterol ratio; NLR, neutrophil to lymphocyte ratio; SIRI/HDL, SIRI/high-density lipoprotein cholesterol count; SIRI × LDL, SIRI × low-density lipoprotein cholesterol count; PIV/HDL, PIV/high-density lipoprotein cholesterol count; PIV × LDL, PIV × low-density lipoprotein cholesterol count.

### 3.4. Risk factor analysis for coronary heart disease

The presence of CHD was used as the dependent variable to assess its association with various factors. Further analysis was performed using multivariate logistic regression to identify independent influencing factors for the occurrence of CHD. The complex indexes with statistically significant associations in the single-factor analysis, along with other variables that showed statistical significance in the single-factor analysis, were included in the multivariate logistic regression analysis. The results demonstrated that SIRI/HDL, SIRI × LDL, PIV/HDL, PIV, SIRI, SII, MHR, and NLR could be considered as independent influencing factors for the occurrence of CHD (Table 5). Among these factors, MHR was found to be a stronger independent risk factor for CHD compared to other complex indexes. Overall, it suggests that SIRI/HDL, SIRI × LDL, PIV/HDL, PIV, SIRI, SII, MHR, and NLR have significant associations with the development of CHD. These factors may serve as valuable indicators for assessing the risk of CHD, with MHR showing the strongest independent association.

**Table 5:** Multi-factor logistic regression analysis of risk factors for CHD.

Indicators	β	Standard error	Wald value	OR (95% CI of OR)	*P*-value
SIRI/HDL	0.442	0.090	23.851	1.556 (1.303–1.857)	<0.001
SIRI × LDL	0.105	0.027	14.684	1.111 (1.053–1.172)	<0.001
PIV/HDL	0.002	0.000	21.754	1.002 (0.996–1.008)	<0.001
PIV × LDL	0.000	0.000	10.999	1.000 (1.000–1.001)	0.001
PIV	0.001	0.000	12.654	1.001 (1.001–1.002)	<0.001
MHR	1.572	0.366	18.480	4.816 (2.352–9.863)	<0.001
NLR	0.177	0.042	17.667	1.194 (1.099–1.296)	<0.001
SII	0.001	0.000	18.881	1.001 (1.000–1.001)	<0.001
SIRI	0.307	0.820	13.884	1.359 (1.157–1.597)	<0.001

PIV, pan-immune-inflammation value; SIRI, systemic inflammation-response index; SII, systemic immune-inflammation index; MHR, monocyte to high-density lipoprotein cholesterol ratio; NLR, neutrophil to lymphocyte ratio; SIRI/HDL, SIRI/ high-density lipoprotein cholesterol count; SIRI × LDL, SIRI × low-density lipoprotein cholesterol count; PIV/HDL, PIV/high-density lipoprotein cholesterol count; PIV × LDL, PIV × low-density lipoprotein cholesterol count.

### 3.5. Comparison of the efficacy of each complex index for the diagnosis of coronary heart disease and ROC curve analysis

The results demonstrated that SIRI/HDL exhibited superior effectiveness in diagnosing coronary heart disease compared to SIRI × LDL, PIV/HDL, PIV × LDL, PIV, SIRI, SII, MHR, NLR, with the highest efficacy observed when the cut-off value for SIRI/HDL was set at 1.015. The sensitivity of SIRI/HDL was found to be 0.667%, specificity was 0.604%, and the area under the curve was 0.680 (95% CI: 0.658–0.701), indicating good diagnostic performance. On the other hand, PIV × LDL demonstrated the highest sensitivity of 85.2%, while PIV/HDL exhibited the highest specificity of 0.623% (Table 6 and Figure 1).

**Table 6:** Comparison of the efficacy of each complex index for the diagnosis of CHD.

Indicators	Optimal cut-off point	Sensitivity (%)	Specificity (%)	Area under the curve	Yoden Index	95% confidence interval
SIRI/HDL	1.015	0.667	0.604	0.680	0.271	0.658–0.701
SIRI × LDL	3.505	0.756	0.469	0.644	0.224	0.623–0.666
PIV/HDL	182.12	0.618	0.623	0.668	0.240	0.647–0.690
PIV × LDL	895.24	0.852	0.359	0.634	0.211	0.612–0.656
PIV	269.95	0.751	0.459	0.647	0.210	0.625–0.669
MHR	0.475	0.732	0.476	0.638	0.208	0.616–0.660
NLR	3.025	0.794	0.403	0.625	0.196	0.603–0.647
SII	654.11	0.813	0.361	0.619	0.173	0.596–0.641
SIRI	1.335	0.766	0.473	0.659	0.239	0.638–0.680

PIV, pan-immune-inflammation value; SIRI, systemic inflammation-response index; SII, systemic immune-inflammation index; MHR, monocyte to high-density lipoprotein cholesterol ratio; NLR, neutrophil to lymphocyte ratio; SIRI/HDL, SIRI/high-density lipoprotein cholesterol count; SIRI × LDL, SIRI × low-density lipoprotein cholesterol count; PIV/HDL, PIV/high-density lipoprotein cholesterol count; PIV × LDL, PIV × low-density lipoprotein cholesterol count.

## 4. Discussion

To the best of our knowledge, the correlation PIV and the severity of coronary artery stenosis has not been explored in previous studies. Furthermore, to gain a deeper understanding, we combined leukocyte subtypes, platelet count, and lipid profile to create complex indexes. This allowed us to analyze the relationship between the severity of coronary artery stenosis and these complex indexes, including SIRI/HDL, SIRI × LDL, PIV/HDL, PIV × LDL, PIV, SIRI, SII, MHR, and NLR, and their predictive value for CHD. There are some key findings. First, we demonstrated that PIV were positively correlated with the severity of coronary artery lesions through a large sample analysis. Second, although PIV is a new inflammatory indicator that can diagnose CHD, its diagnostic ability is not as good as that of the previous inflammatory indicator SIRI. Third, by combining the inflammatory indicator SIRI with HDL, we derived the SIRI/HDL index, which may enhance the diagnostic accuracy of CHD. Forth, compared with single inflammatory indicators, the complex index obtained by combining them with HDL can improve the diagnostic ability of CHD. In contrast, the diagnostic ability of the complex index obtained by combining them with LDL becomes weaker.

The incidence of CHD has been increasing due to changes in lifestyle and societal and economic development. Additionally, there is a concerning trend of CHD occurring at younger ages (8). AS we know, CHD is considered a chronic inflammatory disease and is a result of the narrowing or obstruction of coronary arteries due to atherosclerosis. It is characterized by the accumulation of lipids, local inflammation, proliferation of smooth muscle cells (SMCs), apoptosis, necrosis, and fibrosis (9). It involves a complex interplay of various inflammatory cells, including monocytes, neutrophils, and lymphocytes, as well as platelets and lipid metabolism (10–15). In addition, arterial stiffness is a recognized predictor of cardiovascular (CV) morbidity and death, and is an early indicator of arteriosclerosis. Inflammatory biomarkers and biomarkers of lipid metabolism were associated with arterial stiffness, a surrogate marker of cardiovascular events, indirectly emphasizing the prophylactic importance of those biomarkers (16, 17). Peripheral blood inflammatory cell counts and their derivatives have gained popularity in clinical practice as easily accessible and cost-effective indicators of chronic low-grade inflammation. These indicators have proven useful in assessing the severity and prognosis of coronary artery disease. Many studies have highlighted the importance of parameters such as MHR, NLR, SII, SIRI in evaluating CHD severity and prognosis (18–26). Moreover, the novel parameter called pan-immune inflammatory value (PIV) has been proposed as a more reliable predictor of clinical outcomes in patients with advanced colorectal cancer (6). Bektas et al. discovered that PIV was able to predict both early and late prognosis in patients with ST-segment elevation myocardial infarction (STEMI) (7). However, relying on a single inflammatory indicator may not provide a complete understanding of inflammation severity. Therefore, early and accurate screening should involve a combination of multiple inflammatory factors and biomarkers of lipid metabolism to predict the severity of the disease more accurately. Based on the above studies, our study focused on the relationship between PIV and the severity of coronary lesions. In addition, while exploring the relationship between PIV and the severity of coronary artery lesions, we boldly combined leukocyte subtypes, platelets and lipids to form complex indexes. To further explore and compare the relationship between SIRI/HDL, SIRI*LDL, PIV/HDL, PIV*LDL, PIV, SIRI, SII, MHR, NLR and the degree of coronary artery stenosis and their predictive value to coronary heart disease.

The Gensini score reflects the plaque load, fully considers the number, location, and degree of stenosis of coronary artery lesions, and can better evaluate the degree of coronary artery lesions. It has been widely used in clinical practice. The SYNTAX score I reflects the type of plaque and the complexity of PCI, and is a guideline for decision-making of revascularization in patients with coronary artery disease, and its predictive value for adverse events has been confirmed in many studies (27). In this study, we assessed the severity of coronary artery lesions using both the Gensini score and the SYNTAX score I in a cohort of 3,201 patients who underwent coronary angiography. Our findings demonstrated a positive correlation between each complex index and the degree of coronary artery stenosis. Notably, SIRI/HDL exhibited the strongest correlation with both the Gensini score and SYNTAX score I. As the level of SIRI/HDL increased, indicating a higher value, the Gensini score also increased, suggesting a more severe coronary artery lesion. Moreover, SIRI/HDL showed superior diagnostic efficacy compared to other composite indices. Using a threshold value of 1.015 for SIRI/HDL, the sensitivity and specificity were determined to be 0.667% and 0.604%, respectively. The area under the curve for SIRI/HDL was 0.680.

In our study, we demonstrated the link between PIV and the severity of coronary artery lesions through a large sample analysis. By combining the inflammatory marker SIRI with HDL, we derived the SIRI/HDL index, which provided a more comprehensive understanding of lipid infiltration, systemic inflammation, and immune system activation in patients with CHD. The blood cell counts and lipid profile, required for calculating SIRI/HDL, can be easily and quickly obtained. Utilizing SIRI/HDL may enhance the diagnostic accuracy of CHD. Additionally, it is worth noting that the complex index PIV × LDL exhibited the highest sensitivity in diagnosing CHD. In clinical practice, it is crucial to recognize the significance of using complex indicators in diagnosing CHD. By employing these complex indexes, we can develop effective strategies for early detection and treatment, benefiting a larger number of patients and improving their prognosis.

## 5. Limitations

In this study, we acknowledged several limitations. One of the most significant limitations was the retrospective design, which was based on the study's retrospective nature. Additionally, our participants were from a single center rather than multiple centers, and the study design was cross-sectional. Furthermore, we only assessed atherosclerosis through coronary angiography, while intravascular ultrasonography and coronary computed tomography might provide more precise information about the extent of coronary atherosclerosis. And due to the existence of multicollinearity among the composite indicators, we conducted multifactorial logistic regression analysis on these indicators independently from the other variables in the study of coronary heart disease. This approach allowed for a more accurate assessment of the impact of the composite indicators on the development of the disease. It was worth noting that some patients may be taking statins, which can affect their TC and LDL levels. However, even with lower LDL levels resulting from medication use, cardiovascular events associated with AS can still occur. This might be due to other lipid compositions contributing to residual risk and ongoing inflammatory reactions during the active AS process (28). While we did discover correlations, it was imperative that additional research could be conducted to verify the clinical relevance of the composite indices of different leukocyte subtypes, lipid, and platelet in relation to AS.

## 6. Conclusion

All complex indexes which we investigated, including PIV, exhibited positive correlations with the severity of coronary artery stenosis. SIRI/HDL, a complex index combing blood cell type and lipid measurements, demonstrated higher diagnostic efficiency for CHD and a significant correlation with the severity of coronary artery stenosis. Incorporating SIRI/HDL into diagnostic protocols may improve the accuracy of CHD diagnosis.

## Data Availability

The raw data supporting the conclusions of this article will be made available by the authors, without undue reservation.
